# Small-molecule inhibitor of intestinal anion exchanger SLC26A3 for treatment of hyperoxaluria and nephrolithiasis

**DOI:** 10.1172/jci.insight.153359

**Published:** 2022-07-08

**Authors:** Onur Cil, Tifany Chu, Sujin Lee, Peter M. Haggie, Alan S. Verkman

**Affiliations:** 1 Department of Pediatrics, Division of Pediatric Nephrology, and; 2 Departments of Medicine and Physiology, UCSF, San Francisco, California, USA.

**Keywords:** Nephrology, Therapeutics, Epithelial transport of ions and water

## Abstract

Nephrolithiasis is a common and recurrent disease affecting 9% of the US population. Hyperoxaluria is major risk factor for calcium oxalate kidney stones, which constitute two-thirds of all kidney stones. SLC26A3 (DRA, downregulated in adenoma) is an anion exchanger of chloride, bicarbonate, and oxalate thought to facilitate intestinal oxalate absorption, as evidenced by approximately 70% reduced urine oxalate excretion in knockout mice. We previously identified a small-molecule SLC26A3 inhibitor (DRA_inh_-A270) that selectively inhibited SLC26A3-mediated chloride/bicarbonate exchange (IC_50_ ~ 35 nM) and, as found here, oxalate/chloride exchange (IC_50_ ~ 60 nM). In colonic closed loops in mice, luminal DRA_inh_-A270 inhibited oxalate absorption by 70%. Following oral sodium oxalate loading in mice, DRA_inh_-A270 largely prevented the 2.5-fold increase in urine oxalate/creatinine ratio. In a mouse model of oxalate nephropathy produced by a high-oxalate low-calcium diet, vehicle-treated mice developed marked hyperoxaluria with elevated serum creatinine, renal calcium oxalate crystal deposition, and renal injury, which were largely prevented by DRA_inh_-A270 (10 mg/kg twice daily). DRA_inh_-A270 administered over 7 days to healthy mice did not show significant toxicity. Our findings support a major role of SLC26A3 in intestinal oxalate absorption and suggest the therapeutic utility of SLC26A3 inhibition for treatment of hyperoxaluria and prevention of calcium oxalate nephrolithiasis.

## Graphical abstract

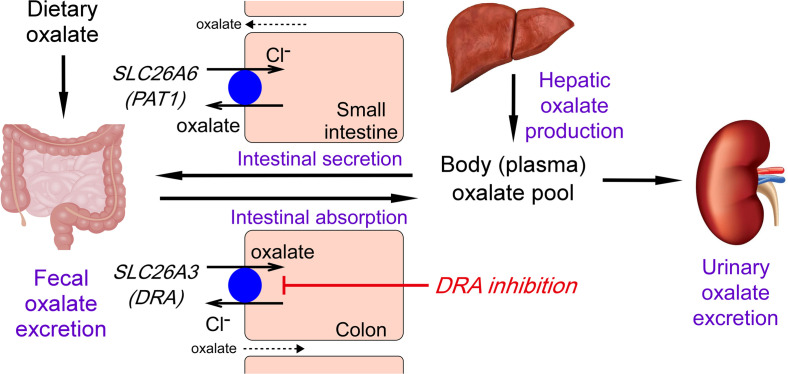

## Introduction

Nephrolithiasis affects at least 9% of individuals living in the US during their lifetime, and its prevalence is increasing (1). More than 80% of kidney stones contain calcium, with calcium oxalate being the primary constituent in at least two-thirds of all kidney stones (2). Nephrolithiasis has a high recurrence rate of approximately 50% in 5 years (3). Current approaches to reduce calcium oxalate stone recurrence include general measures, such as increasing fluid intake and dietary salt and oxalate restriction. Additionally, thiazide diuretics and potassium citrate are used depending on urinary metabolic abnormalities, such as hypercalciuria and/or hypocitraturia. There are no approved drugs for treatment of hyperoxaluria, a major and common risk factor for calcium oxalate nephrolithiasis and recently recognized risk factor for progression of chronic kidney disease (CKD) (4).

Oxalate is a divalent anion with no known biological function in humans. Oxalate is present in many foods and is also generated by liver as a waste product of ascorbic acid, purine, amino acid, and carbohydrate metabolism (5). The majority (~90%) of oxalate is excreted in the urine, in which it can form insoluble calcium oxalate crystals, leading to calcium oxalate nephrolithiasis, nephrocalcinosis, and CKD (5, 6). Under normal conditions, dietary oxalate contributes approximately 30%–40% of the urinary oxalate burden, with the remaining oxalate produced by the liver (7, 8). Colonic oxalate absorption is greatly increased in conditions associated with fat malabsorption, including bariatric surgery, inflammatory bowel disease, intestinal resection, and pancreatic insufficiency, resulting in “enteric hyperoxaluria” (9). Enteric hyperoxaluria occurs in up to 74% of patients undergoing gastric bypass for obesity (9, 10) and 60% of individuals with cystic fibrosis (due to pancreatic insufficiency), resulting in a 3-fold increased incidence of nephrolithiasis compared with that in the general population (11, 12). Because of the proposed important role of the intestines in oxalate handling, we postulated that pharmacological modulation of intestinal oxalate transport may be beneficial for treatment of enteric and idiopathic hyperoxaluria and prevention of calcium oxalate nephrolithiasis.

Oxalate is transported in the gastrointestinal tract through transcellular and paracellular pathways, though the exact contribution of each pathway, as well as the ion transporters involved, has been unclear, in part, due to the lack of selective pharmacological inhibitors. Transcellular oxalate transport is thought to be mediated by the SLC26A family anion exchangers SLC26A6 (also known as PAT1) in small intestine and SLC26A3 (originally named downregulated in adenoma [DRA]) in colon, with net oxalate secretion in small intestine and net absorption in colon under normal conditions (13). In mice, SLC26A6 loss of function impairs intestinal oxalate secretion and causes nephrolithiasis (14), whereas SLC26A3 loss of function impairs colonic oxalate absorption and reduces urinary oxalate excretion by 70% (15). These findings provide evidence supporting an important role of SLC26A family anion exchangers and transcellular oxalate transport in intestinal oxalate handling. However, earlier in vitro studies in transfected cell models reported low SLC26A3-mediated oxalate transport compared with chloride or bicarbonate transport (16, 17). Thus, there is uncertainty about whether SLC26A3 mediates physiologically significant oxalate transport and, therefore, whether SLC26A3 inhibition would be therapeutically beneficial for conditions associated with hyperoxaluria.

**Figure 1:**
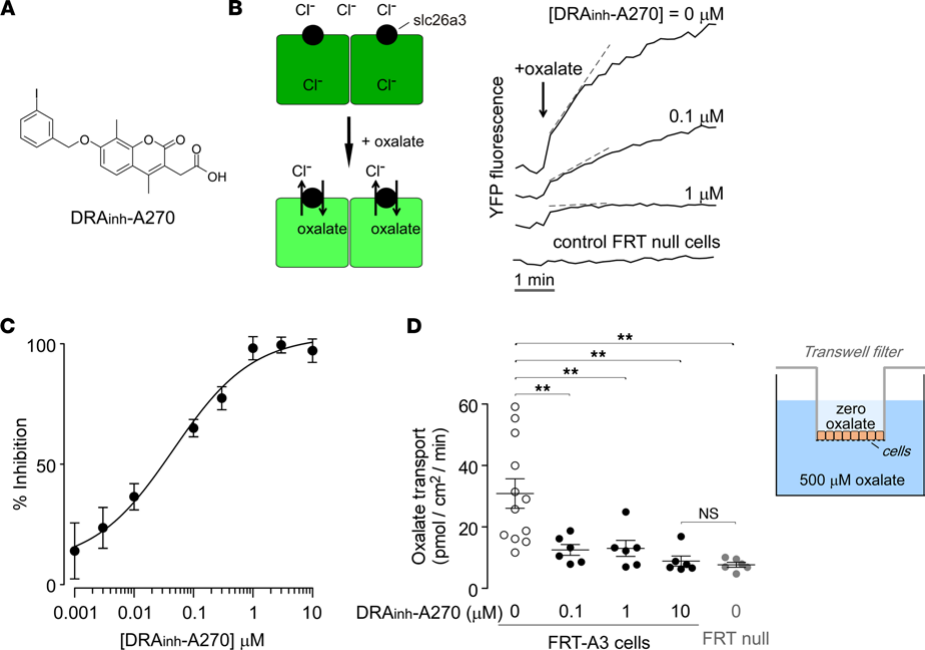
DRA_inh_-A270 inhibits SLC26A3-mediated oxalate transport. (**A**) Chemical structure of DRA_inh_-A270. (**B**) Oxalate/chloride exchange assay (left). Extracellularly added oxalate drives oxalate/chloride exchange and the consequent increase in YFP fluorescence (reduced quenching of cytoplasmic YFP by chloride). Representative curves of YFP fluorescence over time in FRT cells expressing SLC26A3 treated with indicated concentrations of DRA_inh_-A270, with FRT-null cells shown as control (right). Dashed lines denote deduced initial slopes. (**C**) Concentration-dependent inhibition of SLC26A3-mediated Cl^–^/oxalate exchange by DRA_inh_-A270. Mean ± SEM, *n* = 4–8 wells per concentration. (**D**) The inset shows a schematic of oxalate transport assay in FRT cells grown on permeable filters, with basolateral-to-apical oxalate gradient (500 μM oxalate in the basolateral solution and 0 oxalate in the apical solution). Oxalate transport rate is plotted for studies done in FRT cells expressing SLC26A3 (FRT-A3) pretreated with indicated concentrations of DRA_inh_-A270 (or DMSO control) for 15 minutes before application of the oxalate gradient. FRT cells not expressing SLC26A3 (FRT-null cells) are shown as controls. Mean ± SEM, *n* = 6–12 wells per condition. One-way ANOVA with post hoc Newman-Keuls multiple comparisons test; **P < 0.01.

We recently identified, by high-throughput screening, first-in-class, selective small-molecule inhibitors of SLC26A3 with nanomolar potency and favorable pharmacokinetic properties suitable for once or twice daily dosing and, based on their antiabsorptive action in colon, efficacy in normalizing stool hydration in a mouse model of constipation (18, 19). Here, we report inhibition of SLC26A3-mediated oxalate transport in cells and mouse intestine by lead candidate DRA_inh_-A270 (compound 4az in ref. 19; structure shown in Figure 1A) and efficacy in reducing urinary oxalate excretion and prevention of renal calcium oxalate crystal deposition and renal injury in mouse models of hyperoxaluria and oxalate nephropathy.

## Results

### DRA_inh_-A270 inhibits SLC26A3-mediated oxalate transport in transfected cells.

Oxalate/chloride exchange was measured in Fischer rat thyroid (FRT) cells expressing SLC26A3 (FRT-A3 cells) and a halide-sensing yellow fluorescent protein (YFP) (Figure 1B, left). Incubation in a chloride-containing buffer results in reduction (quenching) of YFP fluorescence. Extracellular addition of a chloride-free solution containing oxalate drives cellular oxalate uptake in exchange for chloride, resulting in increased (dequenched) YFP fluorescence, with little fluorescence increase seen in FRT-null cells lacking SLC26A3 (Figure 1B, right). DRA_inh_-A270 inhibited this SLC26A3-mediated oxalate/chloride exchange with IC_50_ ~ 60 nM (Figure 1C). For these experiments, a supraphysiological oxalate concentration (93 mM) was used, as necessitated by the fluorescent indicator method. In order to determine the effects of DRA_inh_-A270 on oxalate transport at physiologically relevant concentrations, transepithelial oxalate transport was measured in FRT-A3 cell monolayers on permeable filters and exposed to a 500 μM oxalate concentration gradient (Figure 1D). FRT-A3 cells had approximately 4.5-fold increased oxalate transport compared with FRT-null cells, and the increased oxalate transport in FRT-A3 cells was largely blocked by DRA_inh_-A270 pretreatment, even at 0.1 μM. Similar studies were done using fluorescein sulfonic acid (FSA) as a marker of paracellular permeability (20, 21). Under conditions in which DRA_inh_-A270 strongly reduced oxalate transport (Supplemental Figure 1A; supplemental material available online with this article; https://doi.org/10.1172/jci.insight.153359DS1) there was no effect on FSA transport (Supplemental Figure 1B) nor was there an effect on transepithelial electrical resistance (TEER, Supplemental Figure 1C). These results suggest that the action of DRA_inh_-A270 is due to inhibition of transcellular, SLC26A3-facilitated oxalate transport.

### DRA_inh_-A270 prevents intestinal oxalate absorption and reduces hyperoxaluria induced by oral sodium oxalate loading.

**Figure 2:**
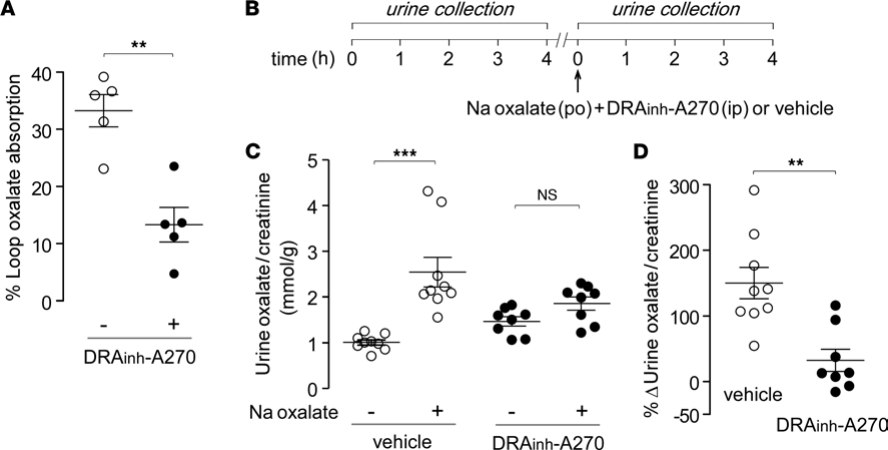
DRA_inh_-A270 blocks intestinal oxalate absorption and prevents hyperoxaluria following acute oral sodium oxalate loading. (**A**) Percentage absorption of luminal oxalate (500 μM, at 0 min) in mouse distal colonic loops at 60 minutes in the presence and absence of 10 μM DRA_inh_-A270. (**B**) Experimental protocol for acute oxalate loading. (**C**) Urine oxalate/creatinine ratio in mice measured in 4-hour urine collections before and after oral gavage of 2.5 μmol/kg sodium oxalate. Mice (CD1 strain) were administered vehicle or DRA_inh_-A270 (10 mg/kg, i.p.) at the time of oral sodium oxalate loading. (**D**) Percentage increase in urine oxalate/creatinine ratio shown in individual mice. Mean ± SEM, *n* = 8–9 mice per group. Unpaired *t* test (**A** and **D**) and paired *t* test (**C**); **P < 0.01, ***P < 0.001.

To investigate DRA_inh_-A270 inhibition of intestinal oxalate transport, experiments were done using closed distal colonic loops in which SLC26A3 is the major apical membrane anion transporter (18, 22). Colonic loops were injected with the solution containing 500 μM sodium oxalate and loop fluid was removed at 60 minutes to quantify remaining oxalate concentration. To avoid confounding effects of fluid absorption, Cl^–^-free solution with amiloride (to block ENaC) was used (Supplemental Figure 2A). In this model, luminal DRA_inh_-A270 reduced oxalate absorption by approximately 70% compared with vehicle control (Figure 2A). To investigate whether the DRA_inh_-A270 effect on in vivo oxalate absorption is related to changes in paracellular permeability, similar experiments were done using mannitol as a marker of paracellular permeability. Under conditions in which DRA_inh_-A270 reduced oxalate absorption by approximately 60%, there was no effect on mannitol absorption (Supplemental Figure 2B).

As another in vivo model, an acute model of hyperoxaluria was established (Figure 2B) in which bolus oral administration of 2.5 μmol/kg sodium oxalate in mice produced an approximately 2.5-fold increase in urine oxalate/creatinine ratio. This increase in urine oxalate/creatinine ratio was reduced by approximately 80% with DRA_inh_-A270 administration (Figure 2, C and D), supporting the conclusion that DRA_inh_-A270 blocks intestinal oxalate uptake in vivo. Absolute oxalate and creatinine concentration data for these studies are provided in Supplemental Figure 2C.

### DRA_inh_-A270 prevents oxalate nephropathy in mice.

**Figure 3:**
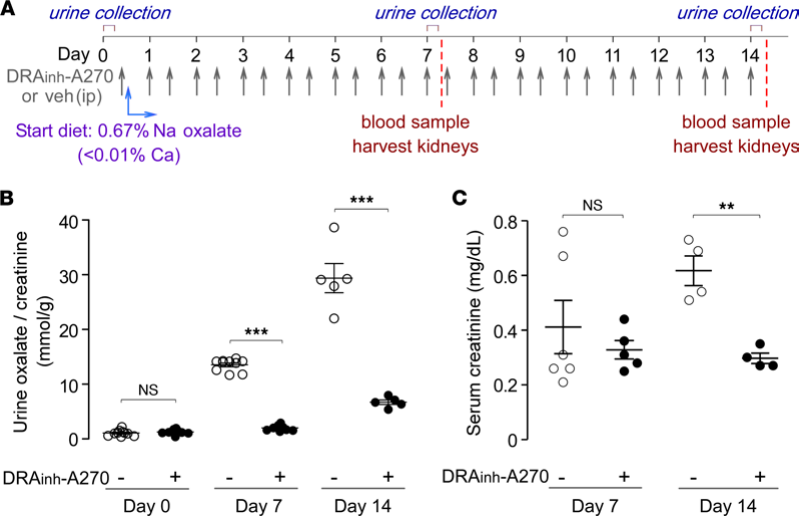
DRA_inh_-A270 prevents oxalate nephropathy in mice on a high-oxalate low-calcium diet. (**A**) Experimental protocol. (**B**) Urine oxalate/creatinine ratio at days 0, 7, and 14 in mice (C57BL/6 strain) treated with DRA_inh_-A270 (10 mg/kg, i.p., twice daily, starting day 0) or vehicle. *n* = 5–10 mice per group per time point. (**C**) Serum creatinine at days 7 and 14 in mice treated with DRA_inh_-A270 or vehicle. Mean ± SEM, *n* = 4–6 mice per group per time point. Unpaired *t* test; **P < 0.01, ***P < 0.001.

A chronic model of hyperoxaluria and oxalate nephropathy in mice was induced using a high-oxalate, low-calcium diet (Figure 3A) in which reduced dietary calcium increases the oral bioavailability of oxalate, resulting in hyperoxaluria and progressive renal injury, as reported previously (23, 24). At days 7 and 14 on the high-oxalate diet, the urinary oxalate/creatinine ratio increased by approximately 12- and 27-fold, respectively, which was largely prevented by DRA_inh_-A270 (10 mg/kg, i.p., twice daily starting at day 0) (Figure 3B). Absolute oxalate and creatinine concentration data for these studies are provided in Supplemental Figure 3. Serum creatinine on day 14 was approximately 2-fold higher in vehicle-treated mice than in DRA_inh_-A270–treated mice (Figure 3C).

**Figure 4:**
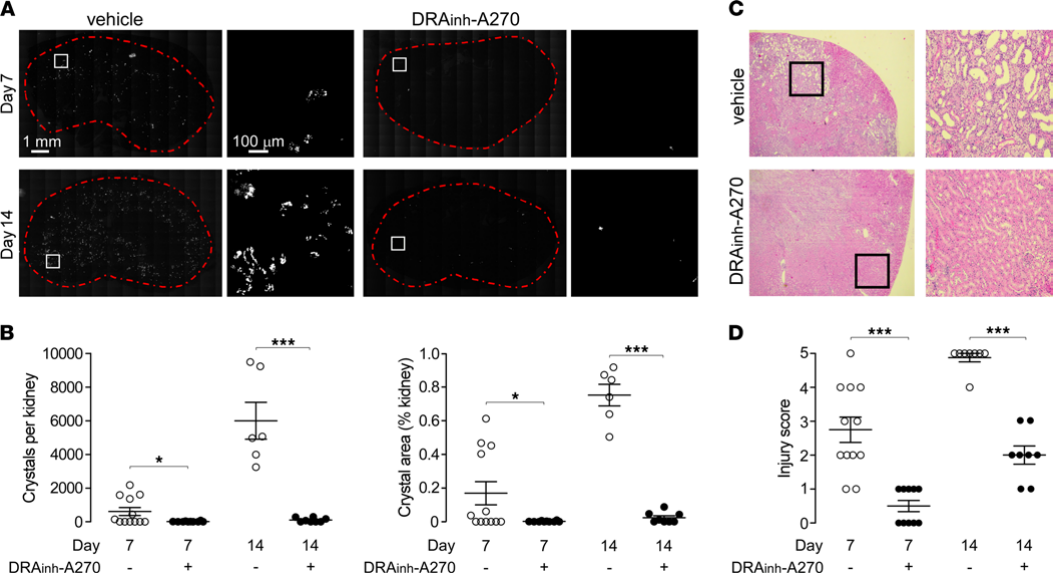
DRA_inh_-A270 prevents renal calcium oxalate deposition and renal injury in the diet-induced oxalate nephropathy model. (**A**) Polarization light microscopy of whole kidney sections (kidney boundary denoted by red dashed lines) in mice fed with high-oxalate diet for 7 or 14 days and treated with vehicle or DRA_inh_-A270 (as per Figure 3A). Boxed regions are presented at higher magnification on the right. Scale bar: 1 mm (first and third column); 100 μm (second and fourth column). (**B**) Total number and total area of calcium oxalate crystals per kidney in mice fed with high-oxalate diet for 7 or 14 days and treated with vehicle or DRA_inh_-A270. *n* = 6–12 kidneys per group per time point. (**C**) H&E-stained kidney sections at day 7 of high-oxalate diet in mice treated with vehicle or DRA_inh_-A270, representative of 10–12 kidneys per group. Boxed regions are presented at higher magnification on the right. 10x magnification (left); 40x magnification (right). (**D**) Renal injury score on days 7 and 14 of the high-oxalate diet in mice treated with vehicle or DRA_inh_-A270. Mean ± SEM, *n* = 8–12 kidneys per group per time point. Unpaired *t* test; *P < 0.05, ***P < 0.001.

Kidneys harvested on days 7 and 14 were examined by polarized light microscopy for calcium oxalate crystal deposition. Progressive crystal deposition was seen in vehicle-treated mice (Figure 4A), which was largely prevented by DRA_inh_-A270 treatment. Quantitative image analysis revealed remarkably fewer calcium oxalate crystals in kidneys of DRA_inh_-A270 -treated mice, as determined by crystal counting and area occupied by crystals (Figure 4B). H&E staining of kidneys showed marked injury in vehicle-treated mice, with characteristic findings of oxalate nephropathy, including tubular necrosis and interstitial inflammation (Figure 4C). The renal injury score was much reduced in the DRA_inh_-A270–treated mice compared with that in vehicle-treated mice (Figure 4D).

### Selectivity, off-target effects, and toxicity.

We previously reported that DRA_inh_-A270 at high concentration does not inhibit anion transport by SLC26A4, SLC26A6, or SLC26A9 (19). SLC26A1 and SLC26A2 are other members of the SLC26A family expressed in intestinal epithelial cells with possible roles in oxalate transport (13). No significant inhibition of slc26a1- or slc26a2-mediated anion transport was found for DRA_inh_-A270 at 10 μM (Supplemental Figure 4). Our original high-throughput screen was done in FRT cells expressing murine slc26a3 (18). Supplemental Figure 5 shows strong inhibition of human SLC26A3 anion transport by DRA_inh_-A270 with 20 nM IC_50_.

Possible off-target effects of DRA_inh_-A270 were investigated by panel screening (Eurofins SafetyScreen44). In this panel, a “hit” is defined as giving more than 50% inhibition at 10 μM and compounds with less than 5% “target hit rate” are considered selective (25, 26). DRA_inh_-A270 had a low (<5%) target hit rate at 10 μM, which suggests good selectivity (Supplemental Table 1). Testing for CYP450 and hERG inhibition also supported DRA_inh_-A270 safety (Supplemental Tables 2 and 3, respectively). Finally, metabolic stability of DRA_inh_-A270 was measured in mouse, rat, and human microsomes. Consistent with sustained therapeutic serum levels after single-dose treatment in mice (19), DRA_inh_-A270 had slow elimination half-life (~200 min) in mouse microsomes (Supplemental Table 4) and showed no metabolism in human microsomes, which supports possible once daily dosing.

To assess in vivo toxicity, mice on a regular diet were administered DRA_inh_-A270 (10 mg/kg twice daily, i.p.) or vehicle for 7 days (Supplemental Figure 6A). As diarrhea and metabolic alkalosis due to chloride depletion are theoretical side effects of SLC26A3 inhibition, we compared body weight (Supplemental Figure 6B) as well as stool output and stool water content (Supplemental Figure 6C) in vehicle- and DRA_inh_-A270 treated mice. No significant differences were found. Further, the DRA_inh_-A270 treatment did not have a significant effect on serum electrolytes, anion gap, serum chemistries, or complete blood count (Supplemental Table 5). After chronic administration, DRA_inh_-A270 concentration in serum, as measured by LC/MS, was 8.3 ± 0.4 μM (Supplemental Figure 6D). DRA_inh_-A270 at 10 μM for 24 hours did not affect cell viability in vitro, as assayed by Alamar Blue (Supplemental Figure 6E).

## Discussion

**Figure 5:**
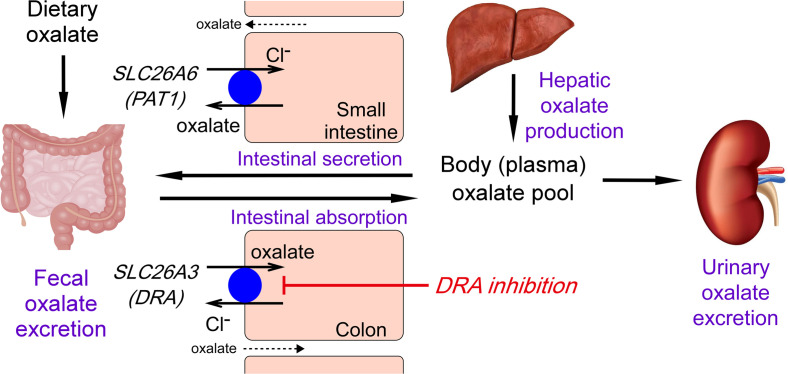
Schematic of oxalate handling in mammals. The systemic oxalate load consists of intestinal absorption of dietary oxalate and hepatic oxalate production. Oxalate is excreted in urine and stool. Oxalate is transported through transcellular and paracellular (dashed arrow) pathways in gut with net secretion in small intestine and net absorption in colon. SLC26A family anion exchangers (SLC26A6 in small intestine and SLC26A3 in colon) play major roles in transcellular oxalate transport. DRA inhibition blocks SLC26A3-mediated transcellular oxalate absorption in colonic epithelium. See Discussion for further explanation.

As the kidneys provide a major excretory pathway for oxalate, they are uniquely affected by the detrimental effects of poorly soluble calcium oxalate, which can result in nephrolithiasis, nephrocalcinosis, and CKD. The gastrointestinal tract both secretes and absorbs oxalate, with net oxalate absorption in colon. SLC26A3 is strongly expressed in colon (13), which is the major site for oxalate absorption in enteric hyperoxaluria (9). The data here support a major role of SLC26A3 in intestinal oxalate absorption, as diagrammed in Figure 5, and provide evidence for pharmacological inhibition of SLC26A3 as a potentially novel approach to reduce urinary oxalate excretion, with potential therapeutic benefits in enteric hyperoxaluria and calcium oxalate nephrolithiasis.

As mentioned in the Introduction, the markedly reduced urinary oxalate excretion in SLC26A3-knockout mice (15) supports a major role of SLC26A3 in intestinal oxalate absorption. There is limited similar data in humans with rare *SLC26A3* mutations (congenital chloride diarrhea [CLD]) (27, 28). In a cohort of humans with CLD (mean age, 22.1 years) the average urine oxalate excretion was reported as 12 mmol/mol creatinine (27), though a healthy control group for comparison was not reported. Based on a normal urinary oxalate excretion of 20–40 mg/d in healthy individuals (28), a 70 kg human with a 24-hour urinary creatinine excretion of 20 mg/kg/d would have a daily urinary oxalate excretion of 19–38 mmol/mol creatinine. Humans with loss-of-function *SLC26A3* mutations, therefore, have an estimated 40%–70% lower urinary oxalate excretion compared with healthy controls, further supporting the potential therapeutic utility of pharmacological SLC26A3 inhibition in hyperoxaluria.

The estimated annual expenditure for management of kidney stone disease is greater than $5 billion in the US, which includes emergency room visits, hospital stays, and procedures. This cost is anticipated to increase owing to increasing prevalence of major risk factors such as obesity and diabetes mellitus (29). In addition, more than 100,000 bariatric surgeries are performed annually in the US, with up to 74% of these patients developing enteric hyperoxaluria (9, 10). Given the high recurrence rate of kidney stones, preventive measures such as modification of diet/fluid intake and citrate supplementation are often used, but with limited success. By reducing intestinal oxalate absorption and, hence, directing oxalate excretion to the stool, SLC26A3 inhibition provides a potentially novel, targeted approach to reducing urinary oxalate excretion in nephrolithiasis and enteric hyperoxaluria.

We anticipate that potential side effects of SLC26A3 inhibition will be minimal because SLC26A3 expression is limited mainly to intestine and prostate (30, 31). One possible side effect is diarrhea due to inhibition of colonic chloride absorption. We reported that SLC26A3 inhibitors increased stool water content in constipated mice with slowed intestinal transit but not in control mice (19), in agreement the unchanged stool water content found here for 7-day SLC26A3 inhibitor treatment. Metabolic alkalosis, which is seen in patients with CLD and knockout mice and occurs secondary to intestinal chloride loss (32), was not seen here with 7-day SLC26A3 inhibitor treatment. We note that diarrhea and metabolic alkalosis in knockout mice and in humans with DRA mutations occur in the setting of chronic and complete SLC26A3 loss of function, which probably accounts for their absence in the 7-day treatment studies. Long-term studies using higher inhibitor doses might produce these side effects. Earlier studies in humans with SLC26A3 loss-of-function mutations showed reduced male fertility (33), which is likely due to CFTR dysregulation caused by impaired interactions with the SLC26A3 STAS domain (34). Histological changes were not seen here in male reproductive tissues in the chronic SLC26A3 inhibitor–treated mice (data not shown). Earlier studies also suggested possible roles of SLC26A3 in tumor suppression (35), intestinal barrier function (36, 37), and inflammation (38, 39), perhaps due to altered intracellular pH. However, intestinal malignancies were not seen in a large series of patients with CLD, with only solitary cases of inflammatory bowel disease reported in the same cohort (40). We did not find histological abnormalities in small intestine or colon in chronic, 7-day inhibitor-treated mice (data not shown). Further preclinical toxicity studies with higher doses and longer treatment times will inform on whether these potential side effects might occur in humans with long-term SLC26A3 inhibitor treatment.

Two biologics are in development for treatment of hyperoxaluria. Recombinant oxalate decarboxylase (ALLN-177 or reloxaliase), which metabolizes oxalate in the intestinal lumen, has shown partial efficacy (~20% reduced daily urine oxalate excretion) when given 3 times daily (41). Recent clinical trials evaluating administration up to 5 times a day showed limited efficacy, with a 22.6% reduction in 24-hour urinary oxalate excretion in the treatment group compared with a 9.7% reduction in the placebo group (42). Another biologic under development is Oxabact, a probiotic containing the oxalate metabolizing bacteria *oxalaobacter formigenes*; however, efficacy was not seen in primary hyperoxaluria in short-term clinical trials (43), and longer-term trials are ongoing (ClinicalTrials.gov NCT03116685). Our approach is the first small-molecule treatment of hyperoxaluria to our knowledge. As DRA_inh_-A270 targets intestinal oxalate absorption rather than oxalate metabolism, its efficacy would not depend on intestinal transit or microbiome, and it can potentially be suitable for once daily oral dosing based on rodent pharmacokinetics (19) and the human microsomal stability data reported here.

We noted several limitations of our proof-of-concept study. Though the high-oxalate diet model reproduces many aspects of enteric hyperoxaluria, such as increased luminal free oxalate, it does not recapitulate other features that may be present, such as increased luminal bile acids, steatorrhea, and intestinal inflammation, which may increase paracellular oxalate permeability (14) and theoretically reduce the efficacy of SLC26A3 inhibition in enteric hyperoxaluria. We found here that DRA_inh_-A270 prevented the development of hyperoxaluria in the setting of increased oxalate bioavailability, an important feature of enteric hyperoxaluria. Testing of SLC26A3 inhibitors in models of hyperoxaluria with steatorrhea and increased luminal bile acids, such as Roux-en-Y gastric bypass in rats (44, 45), may be informative. The primary experimental outcome in the animal models reported here is urinary oxalate excretion, which is a major risk factor for calcium oxalate nephrolithiasis. We note that there are no available animal models using calcium oxalate nephrolithiasis as the primary experimental outcome, perhaps due to inability to cause stones in the collecting system of animals. For this reason, available animal models (such as RYGB in rats and primary hyperoxaluria in mice) use urine oxalate excretion and/or renal crystal deposition as the primary outcome (44–46), as used in the current study. The efficacy of new nephrolithiasis therapeutic candidates, such as DRA inhibitors, will ultimately require human clinical testing using kidney stone recurrence as an endpoint. Finally, our study demonstrates efficacy of DRA inhibition in the presence of relatively high intestinal oxalate concentrations, which is relevant to enteric hyperoxaluria. The efficacy of DRA inhibition on urinary oxalate excretion at lower oxalate concentrations in the intestine (such as mice on a regular oxalate diet) might be informative regarding the potential efficacy of DRA inhibitors in idiopathic hyperoxaluria.

We note that DRA_inh_-A270 treatment greatly reduced but did not completely prevent the signs of oxalate nephropathy, which may be due to residual paracellular oxalate permeability. Earlier in vitro studies in transfected cell models using radiolabeled oxalate showed relatively low SLC26A3-mediated oxalate transport compared with chloride or bicarbonate (16, 17). Thus, there has been uncertainty about whether SLC26A3 mediates physiologically significant oxalate transport or has indirect effects on oxalate handling, such as alteration in electrochemical driving forces for oxalate transport. However, data in ex vivo mouse intestine showed greatly impaired oxalate absorption in SLC26A3 deficiency, accounting for the greatly reduced urinary oxalate excretion (15). The results herein with transfected cells and colonic loops support these prior findings and implicate a major role for SLC26A3 in transcellular intestinal oxalate transport in vivo. Finally, we note that the toxicity data reported herein are limited in scope, as they do not include industry-standard long-term studies in multiple species with high-dose treatment.

The efficacy data in hyperoxaluria models, as well as the selectivity and safety data, support further the development of DRA_inh_-A270 as a drug candidate, though we recognize that long-term safety studies in animals will be required prior to human clinical testing. The efficacy studies done here in mice involved i.p. inhibitor administration in which DRA_inh_-A270 likely reaches its target, SLC26A3, at the apical membrane of colonic epithelial cells, by delivery from the blood. Prior studies supported DRA_inh_-A270 action at the cytoplasmic surface of SLC26A3 and indicated good DRA_inh_-A270 cell permeability and oral bioavailability (19). A systemic absorption mechanism is predicted to produce sustained therapeutic DRA_inh_-A270 concentrations at its target.

In conclusion, we found that pharmacological inhibition of colonic anion exchanger SLC26A3 by a selective, small-molecule inhibitor was effective in reducing intestinal oxalate absorption and preventing renal calcium oxalate deposition and injury in animal models of hyperoxaluria, supporting its potential utility as a first-in-class therapy for enteric hyperoxaluria and calcium oxalate nephrolithiasis.

## Methods

### Mice.

Wild-type CD1 and C57BL/6 mice were bred in the UCSF Laboratory Animal Resource Center and housed in communal cages with standard rodent chow (PicoLab Mouse Diet 20, LabDiet) and water available ad libitum.

### Chemicals.

Chemicals were purchased from MilliporeSigma, unless indicated otherwise. DRA_inh_-A270 was synthesized and purified as described previously (19).

### Cell culture.

FRT and HEK293 cells were obtained from the UCSF Cystic Fibrosis Drug Discovery Core Center.

### Cellular oxalate transport assay.

FRT cells stably expressing murine SLC26A3 and halide-sensitive YFP EYFP-H148Q/I152L/F46L (18) were washed 3 times with PBS (310 mOsm/kg H_2_O, pH 7.4: 136.8 mM NaCl, 2.7 mM KCl, 8 mM Na_2_HPO_4_, 1.46 mM KH_2_PO_4_, 0.9 mM CaCl_2_, 0.5 mM MgCl_2_) and then incubated for 15 minutes at 37°C with PBS containing 0.1% DMSO and specified concentrations of DRA_inh_-A270 (50 μL per well). Each well was then injected with 200 μL oxalate buffer (310 mOsm/kg H_2_O, pH 7.4: 93 mM sodium oxalate, 1.35 mM potassium oxalate, 8 mM Na_2_HPO_4_, 1.46 mM KH_2_PO_4_), and fluorescence was measured every 10 seconds for 10 minutes using a plate reader (excitation 485 nm, emission 520 nm). YFP-expressing FRT-null cells were used as negative control. The rate of Cl^–^/oxalate exchange was computed as the initial curve slope (between 60 and 150 s, oxalate buffer injected at 50 s) as calculated by monoexponential regression using GraphPad Prism software.

### Transwell oxalate transport studies.

FRT cells stably expressing murine SLC26A3 (FRT-A3) and FRT-null cells were grown on Transwell permeable polycarbonate filters with 0.4 μM pore size (Corning Inc., product code 3401) and used after they formed an electrically tight monolayer (transepithelial resistance, >500 Ω cm^2^). TEER was measured using a Millicell-ERS Resistance System (MilliporeSigma) with dual-electrode volt-ohmmeter. Net TEER (ohms/cm^2^) was calculated by subtracting the resistance of cell-free media from measured resistance (21). After washing with PBS, cells were incubated with DRA_inh_-A270 (or DMSO control) for 15 minutes in PBS. Then, the basolateral compartment was filled with 600 μL PBS containing 500 μM sodium oxalate, and the apical compartment was filled with 225 μL PBS (oxalate free). Both the basolateral and apical solutions contained specified concentrations of DRA_inh_-A270 and 10 mM glucose. In pilot studies, we verified that the increase in apical oxalate concentration was linear over at least 60 minutes. After incubation of cells for 60 minutes at room temperature, 150 μL of the apical solution was removed and injected into an ion chromatography system (Dionex Aquion, Thermo Fisher Scientific) to quantify oxalate concentration. A standard curve of signal versus oxalate concentration was generated by adding known amounts of sodium oxalate to aqueous solutions. Oxalate concentrations in test samples were calculated by integrating the area under the oxalate peak. The ion chromatography system had linear sensitivity for oxalate (*R*^2^ = 0.9997) between 0 and 1 mM concentrations (Supplemental Figure 7). In some experiments 200 μg/mL (~420 μM) FSA (Thermo Fisher Scientific) was added to the basolateral solution to study effects of DRA_inh_-A270 on paracellular permeability (20, 21). After 60 minutes, FSA concentration in the apical solution was quantified using a fluorescence plate reader (excitation wavelength 495 nm, emission wavelength 521 nm).

### Closed-loop measurements of oxalate absorption.

CD1 mice were given free access to 5% dextrose in water but no solid food for 48 hours and treated rectally with mineral oil (500 μL twice daily, final dose 16 hours before surgery) during this period to evacuate the colon. Closed distal colonic loops (length, ~2 cm; 1 loop per mouse) were isolated as described previously (18, 20). Loops were injected with 100 μL Cl^–^-free HEPES-buffered saline (pH 7.4: 137 mM Na gluconate, 4.5 mM K gluconate, 10 mM HEPES, 1 mM Ca gluconate, and 1 mM Mg gluconate) containing 500 μM Na oxalate, 20 μM amiloride (to block fluid absorption) and 10 μM DRA_inh_-A270 (or DMSO control). In some experiments, mannitol (500 μM) was added to the loop fluid as a paracellular permeability marker (47). Colonic loops were removed at 60 minutes and luminal fluid was collected with a syringe. This fluid was centrifuged at 4000 g for 15 minutes and 50 μL of the supernatant was diluted in 950 μL water. The diluted mixture was filtered using supor membrane syringe filter with 0.45 μM pore size (Pall Corp., product ID 4485) and used to quantify oxalate concentration by ion chromatography. Mannitol concentration in the loop fluid was quantified using a colorimetric assay per manufacturer’s instructions (MilliporeSigma).

### Mouse model of acute hyperoxaluria.

After overnight fasting, CD1 mice (age 8–12 weeks) were placed in metabolic cages for collection of baseline urine over 4 hours. Our CD1 mouse colony has an average whole-gut transit time of 3 hours (48–50), and so a 4-hour urine collection was done to give sufficient time for the gavaged oxalate to be delivered and absorbed in the gut. The same mice were then given 2.5 μmol/kg sodium oxalate (250 μM in water, 0.1 mL per 10 g body weight) by oral gavage and administered DRA_inh_-A270 (10 mg/kg, in PBS containing 5% DMSO and 20% 2-hydroxypropyl-β-cyclodextrin, 0.1 mL per 10 g body weight) or vehicle (i.p.) at the same time. The DRA_inh_-A270 dose was chosen based on prior pharmacokinetics data (19). Mice were placed in metabolic cages again, and urine was collected for the next 4 hours and 6 N HCl was immediately added (20 μL per mL urine) to ensure oxalate stability. Oxalate was measured in urine using a colorimetric assay, and creatinine was measured by BioAssay Systems using a modified Jaffe method.

### Mouse model of diet-induced oxalate nephropathy.

C57BL/6 mice (age 8–12 weeks, male and female) were used, as reported in earlier studies that established the diet-induced oxalate nephropathy model (23, 24). Mice were placed in metabolic cages on day 0 to collect urine over 3 hours. Mice were then started on a high-oxalate, low-calcium diet (0.67% Na oxalate, <0.01% Ca) (20, 21) purchased from Envigo (product code TD.110105) and administered DRA_inh_-A270 (10 mg/kg i.p., twice daily) or vehicle. On day 7, 3-hour urine was collected and half of the mice in each group were euthanized to harvest kidneys and collect blood. The remaining mice were continued on the high-oxalate diet (with DRA_inh_-A270 or vehicle treatments) until day 14, at which time 3-hour urine was collected, followed by blood collection and kidney harvesting. Oxalate and creatinine were measured in urine samples as described above.

### Renal histology and polarized light microscopy.

Both kidneys from each mouse were fixed in 4% paraformaldehyde followed by paraffin embedding. Kidney sections (thickness 4 μm) were cut through the hilum in order to visualize the cortex, medulla, and papilla in the same section. Sections were stained with H&E to assess renal injury using a semiquantitative tubular injury score (24) based on the percentage area (cortex and medulla) showing tubular necrosis and interstitial inflammation, graded as follows: 0, <10%; 1, 11%–20%; 2, 21%–40%; 3, 41%–60%; 4, 61%–80%; 5, >80%. The deidentified slides were read and scored by the same person blindly. The same kidney sections were imaged using polarization microscopy (22) (Nikon TI microscope with ×10 0.3 NA objective, Nikon Inc.) in which whole sections were scanned, and images were stitched using ImageJ software (NIH). Initial studies showed that pixel intensity thresholding using 1600 arbitrary intensity units captured more than 95% of crystals with minimal background, allowing automated determination of the total number and area of crystals, as defined as spots with 4 or more adjacent bright pixels above threshold.

### In vitro toxicity.

FRT cells were cultured for 48 hours at 37°C in black 96-well Costar microplates with clear plastic bottoms and incubated for 24 hours with 10 μM DRA_inh_-A270, 0.1% DMSO (vehicle control) or 20% DMSO (positive control). In vitro cytotoxicity was measured by Alamar Blue assay according to the manufacturer’s instructions (Thermo Fisher Scientific).

### In vivo toxicity.

CD1 mice (age 8–12 weeks, male) were administered DRA_inh_-A270 (10 mg/kg, i.p.) or vehicle twice daily for 7 days. At day 6, mice were placed in metabolic cages individually after DRA_inh_-A270 or vehicle treatment, and stool samples were collected for 3 hours to determine stool weight, number of pellets, and water content as described previously (49, 50). On day 7, 3 hours after the final treatment, mice were anesthetized with isoflurane, and blood was collected by cardiac puncture. Complete blood count was analyzed in whole blood using HEMAVET 950FS (Drew Scientific Inc.). Serum was separated by centrifugation at 4000 g for 20 minutes at 24°C for analysis of chemistries by Idexx Laboratories Inc., using a Beckman Coulter automated analyzer. Reproductive tissues, ileum, and colon were harvested and processed by paraffin embedding and H&E staining.

### Selectivity and safety studies.

The effect of DRA_inh_-A270 on anion transport by slc26a1 (murine), slc26a2 (murine), and SLC26A3 (human) was measured in HEK293 cells that were either transiently transfected with a plasmid to coexpress halide-sensitive YFP and slc26a1 or slc26a2 as described previously (22) or infected to express SLC26A3 and halide-sensitive YFP (18). hERG activity, CYP450 inhibition, and microsomal stability studies were performed by ChemPartner Inc. hERG activity (*n* ≥ 2 per condition) was measured by manual patch-clamp in Chinese hamster ovary cells stably transfected with hERG, and cisapride (0.1 μM) was used as positive control. CYP450 activity was determined for the isoforms 1A2, 2B6, 2C8, 2C9, 2C19, 2D6, and 3A4 in human liver microsomes (Corning) in the presence and absence of 10 μM DRA_inh_-A270 (*n* = 2 per condition). The following substrates were used for each isoform: CYP1A2, phenacetin (30 μM); CYP2C9, diclofenac (10 μM); CYP2C19, S-mephenytoin (35 μM); CYP3A4, midazolam (5 μM) and testosterone (80 μM); CYP2D6, bufuralol (10 μM); CYP2C8, paclitaxel (10 μM); and CYP2B6, bupropion (70 μM). The positive controls included the following: CYP1A2, α-naphthoflavone; CYP2C9, sulfaphenazole; CYP2C19, omeprazole; CYP3A4, ketoconazole; CYP2D6, quinidine; CYP2C8, nicardipine; and CYP2B6, clopidogrel. Microsomal stability was done in mouse, rat, and human liver microsomes (Corning) with a final liver microsomal protein concentration of 0.5 mg/mL. DRA_inh_-A270 was incubated in the reaction mixture with NADPH for 0, 5, 15, 30, and 45 minutes at 37°C in duplicate. The DRA_inh_-A270 concentration was measured at each time point by LC-MS/MS for computation of in vitro elimination half-life. Ketanserin was used as reference compound.

### Statistics.

Data are reported as mean ± SEM. Studies with 2 groups were analyzed using 2-tailed Student’s *t* test; for 3 or more groups, 1-way ANOVA and post hoc Newman-Keuls multiple comparisons test was used. *P* values of less than 0.05 were considered statistically significant.

### Study approval.

Animal experiments were approved by the UCSF Institutional Animal Care and Use Committee and were performed in adherence to the NIH *Guide for the Care and Use of Laboratory Animals* (National Academies Press, 2011).

## Author contributions

OC developed the original idea for this study. OC, PMH, and ASV designed the experiments. OC, TC, SL, and PMH performed the experiments and analyzed the data. OC wrote the paper. ASV and PMH revised the paper. All authors approved the final version of the manuscript.

## Supplementary Material

Supplemental data

## Version 1 05/24/2022

In-Press Preview

## Version 2 07/08/2022

Electronic publication
