# Supplementary material for: Small-molecule inhibitor of intestinal anion exchanger SLC26A3 for treatment of hyperoxaluria and nephrolithiasis
Source: JCI Insight. 2022 Jul 8;7(13):e153359. doi: 10.1172/jci.insight.153359 (PMC9310519; doi:10.1172/jci.insight.153359)
Supplement: Supplemental data [file jciinsight-7-153359-s107.pdf]

**Supplementary Material Table of Contents**

Supplementary Figure 1. Effects of $DRA_{inh}$-A270 on oxalate and fluorescein sulfonic acid (FSA) transport, and electrical resistance.

Supplementary Figure 2. Loop fluid volume in the intestinal oxalate absorption study, effects of $DRA_{inh}$-A270 on mannitol transport, and urine oxalate and creatinine concentrations in the acute oxalate loading study.

Supplementary Figure 3. Urine oxalate and creatinine concentrations in the high-oxalate diet study.

Supplementary Figure 4. $DRA_{inh}$-A270 selectivity against slc26a1 and slc26a2.

Supplementary Figure 5. $DRA_{inh}$-A270 inhibition of human SLC26A3.

Supplementary Figure 6. Toxicity and off-target studies in mice.

Supplementary Figure 7. Oxalate standard curve in aqueous solution.

Supplementary Table 1. Eurofins SafetyScreen44 panel results for $DRA_{inh}$-A270

Supplementary Table 2. CYP450 inhibition of $DRA_{inh}$-A270 showing mean inhibition for each isoform.

Supplementary Table 3. hERG activity.

Supplementary Table 4. Microsomal stability of $DRA_{inh}$-A270 in mouse, rat and human liver microsomes.

Supplementary Table 5. CBC, serum electrolytes, anion gap and chemistries in mice (on regular diet) treated with $DRA_{inh}$-A270 (10 mg/kg) or vehicle twice daily for 7 days.

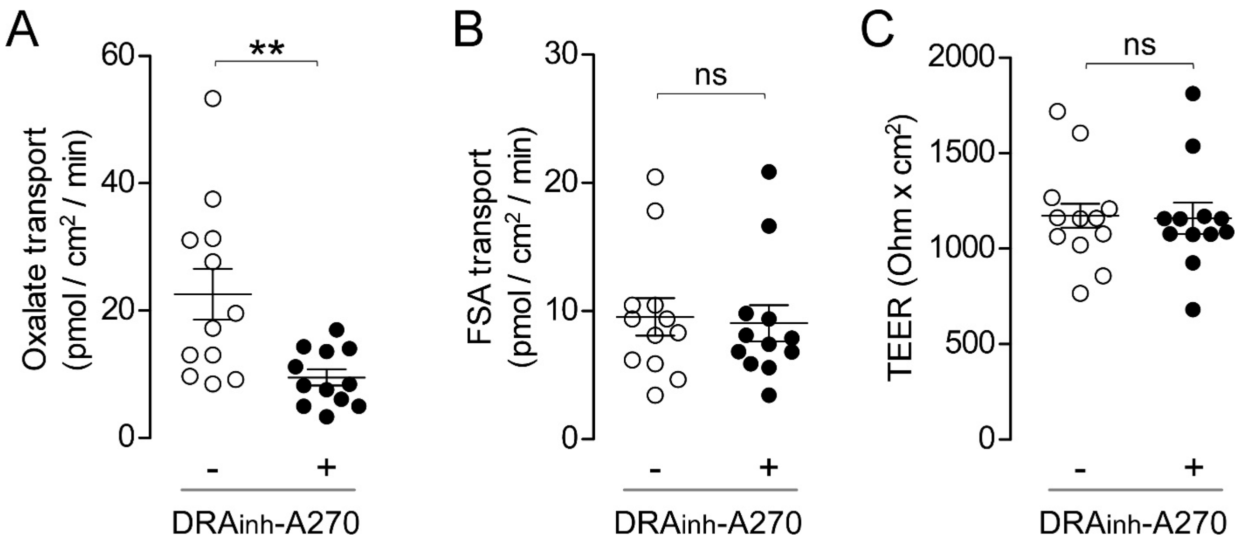


**Supplementary Figure 1. Effects of DRA$_{inh}$-A270 on oxalate and fluorescein sulfonic acid (FSA) transport, and electrical resistance.** Oxalate (**A**) and FSA (**B**) transport rates in FRT cells expressing SLC26A3 (FRT-A3) pretreated with 10 μM DRA$_{inh}$-A270 (or DMSO control) for 15 min prior to application of a 500 μM oxalate and 200 μg/mL (~420 μM) FSA gradient (basolateral-to-apical), as shown in Fig. 1D. **C.** Transepithelial electrical resistance (TEER) in cells treated with 10 μM DRA$_{inh}$-A270 or DMSO control. Mean ± S.E.M., n=12 wells per group. Student's t-test, **p<0.01, ns: not significant.

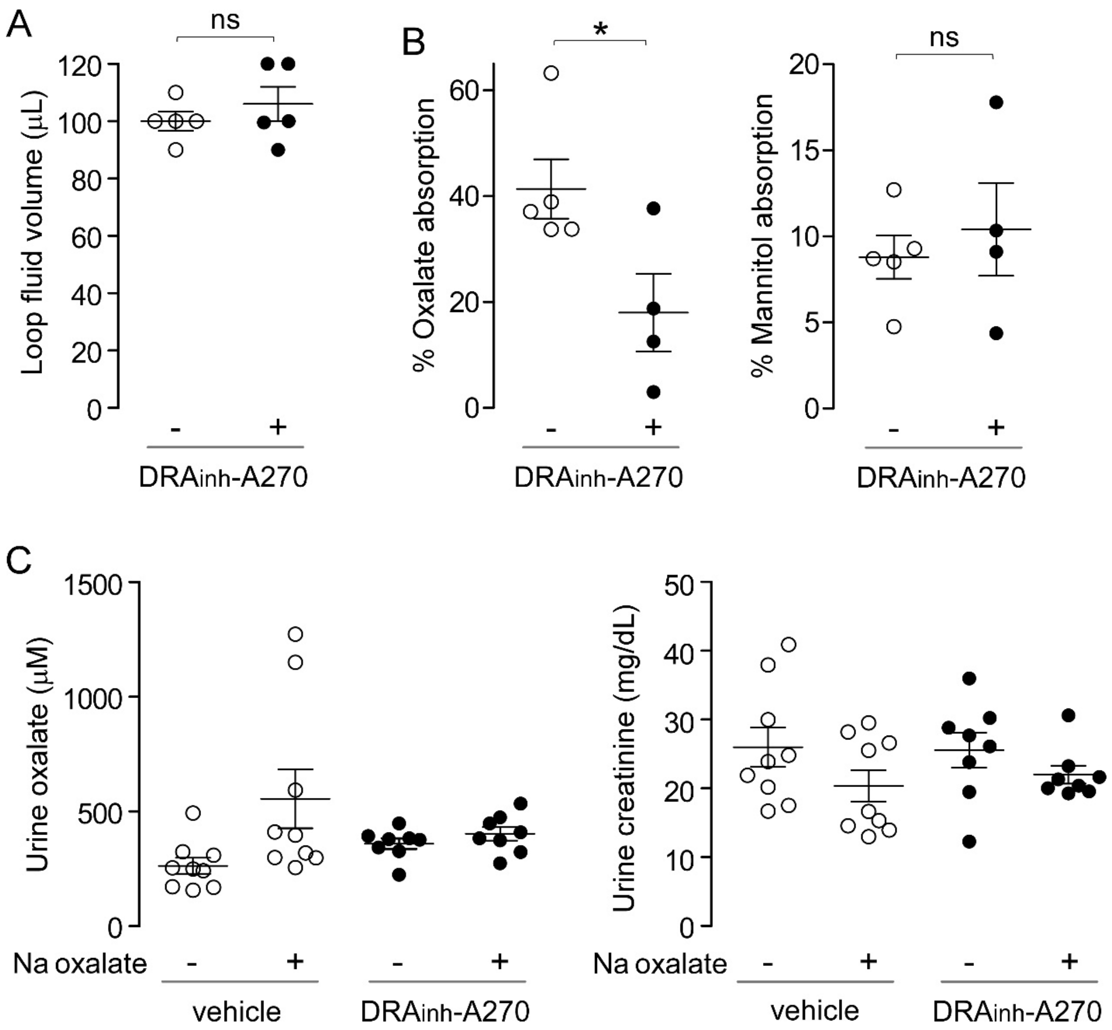


**Supplementary Figure 2. Loop fluid volume in the intestinal oxalate absorption study, effects of DRA$_{inh}$-A270 on mannitol transport, and urine oxalate and creatinine concentrations in the acute oxalate loading study. A.** Loop fluid volume at 60 min in mouse distal colonic loops injected at 0 min with 100 μL of $Cl^-$-free HEPES-buffered saline containing 500 μM sodium oxalate (+20 μM amiloride) with and without 10 μM DRA$_{inh}$-A270. N=5 loops per group. **B.** Percentage absorption of luminal oxalate and mannitol (500 μM each, at 0 min) in mouse distal colonic loops at 60 min in the presence and absence of 10 μM DRA$_{inh}$-A270. N=4-5 loops per group. **C.** Urine oxalate (left) and creatinine (right) concentration in mice (CD1 strain) before and after oral Na oxalate loading (2.5 μmol/kg) and treated with DRA$_{inh}$-A270 (10 mg/kg, ip) or vehicle at the time of oxalate loading. N=8-9 mice per group. Mean ± S.E.M., Student's t-test, *$p<0.05$, ns: not significant.

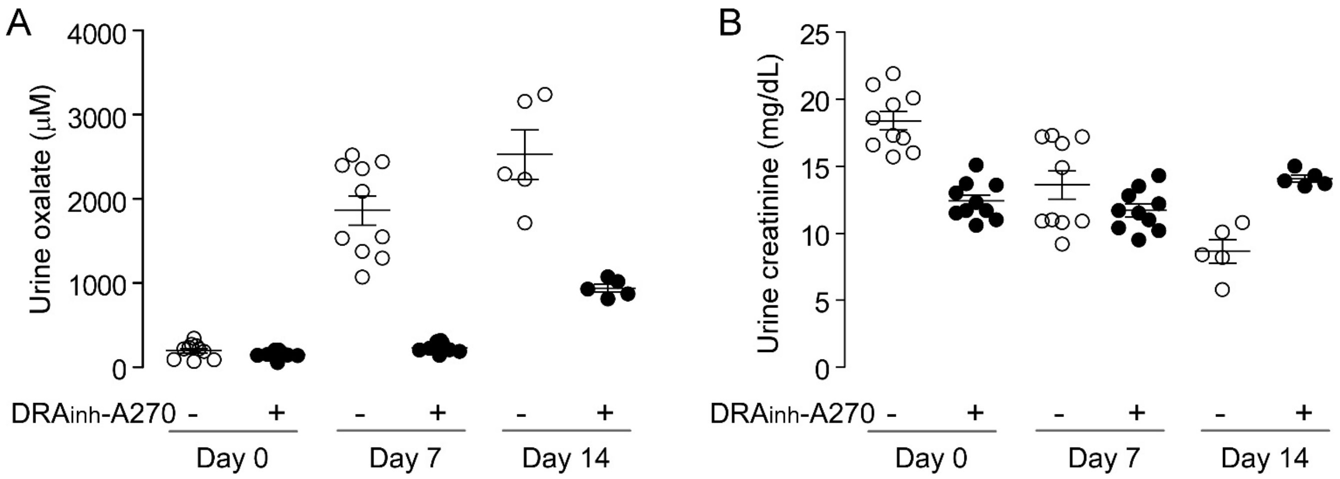


**Supplementary Figure 3. Urine oxalate and creatinine concentrations in the high-oxalate diet study. A.** Urine oxalate concentration in mice (C57BL/6 strain) on Days 0, 7 and 14 of the high-oxalate, low-calcium diet and treated with $DRA_{inh}$-A270 (10 mg/kg, ip, BID) or vehicle. **B.** Urine creatinine concentration in mice on Days 0, 7 and 14 of the high-oxalate, low-calcium diet and treated with $DRA_{inh}$-A270 (10 mg/kg, ip, BID) or vehicle. Mean ± S.E.M., N=5-10 mice per group per time point.

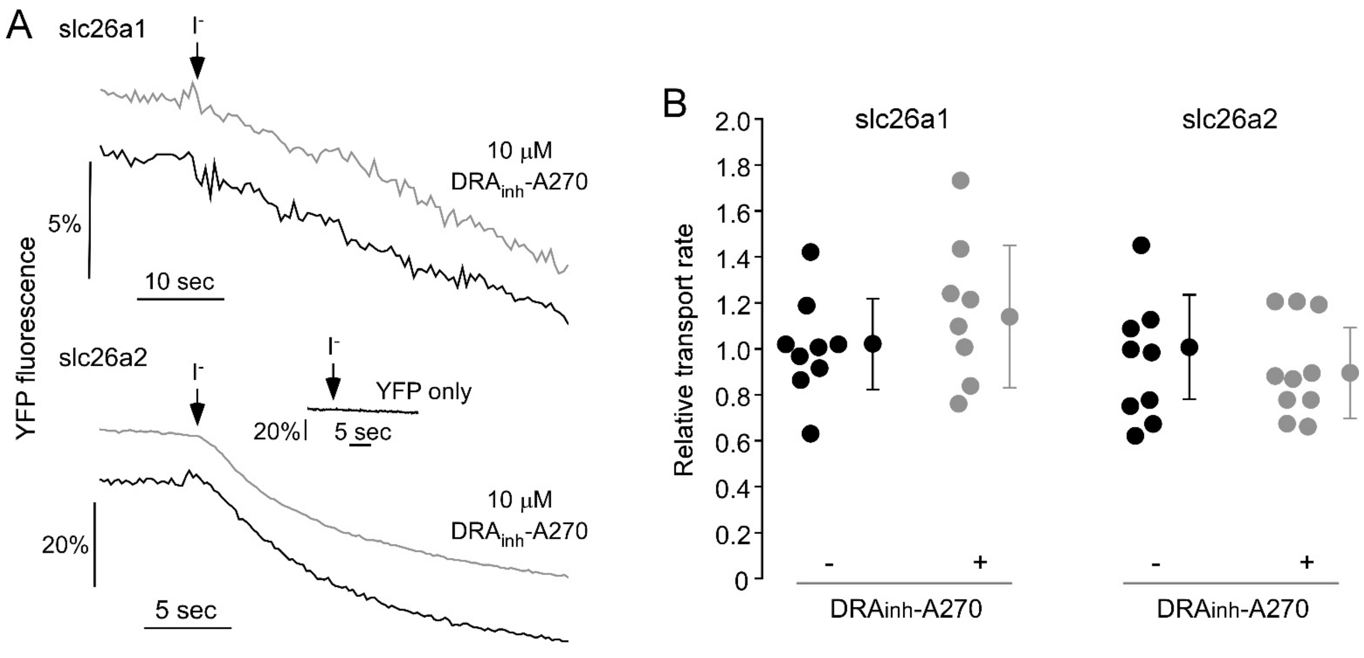


**Supplementary Figure 4. DRA$_{inh}$-A270 selectivity against slc26a1 and slc26a2. A.** 293 T/17 cells were transiently transfected with a plasmid to co-express slc26a1 or slc26a2 and a halide-sensitive YFP. $Cl^-/I^-$ exchange activity shown without and with 10 µM DRA$_{inh}$-A270. **B.** Summary of relative transport activity for slc26a1 and slc26a2 without and with DRA$_{inh}$-A270. N=8-10 experiments per condition, mean ± S.E.M, differences not significant.

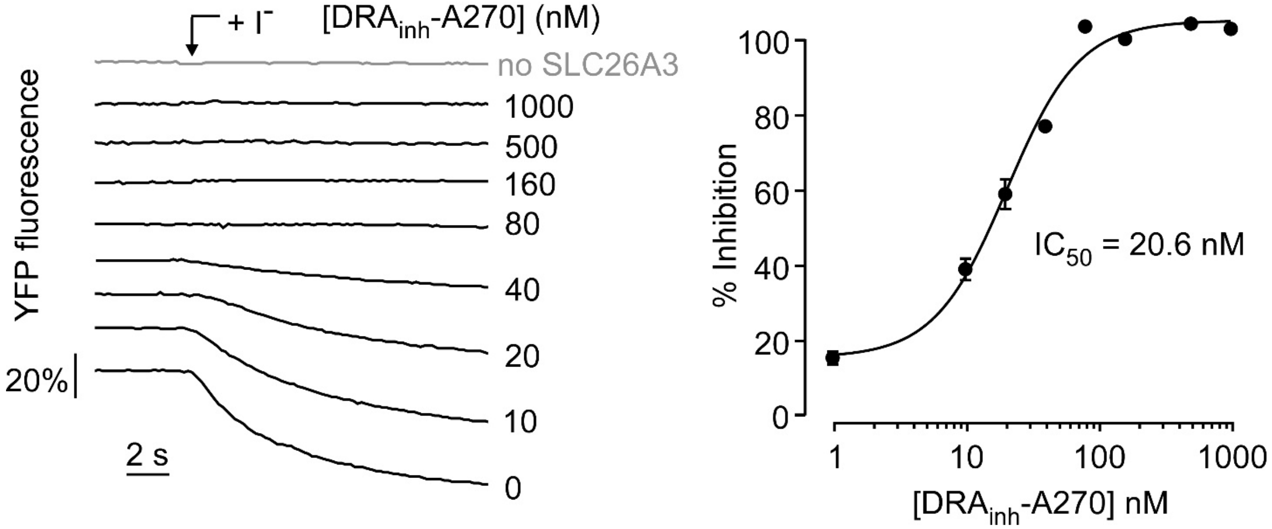


**Supplementary Figure 5. $DRA_{inh}$-A270 inhibition of human SLC26A3.** *(left)* 93 T/17 cells were transiently transfected to co-express human SLC26A3 and a halide-sensitive YFP. YFP fluorescence shown as a measure of $Cl^-/I^-$ exchange in the presence of indicated concentrations of $DRA_{inh}$-A270. The top curve shows FRT-null cells as negative control. *(right)* Summary data for $DRA_{inh}$-A270 inhibition of human SLC26A3. Mean ± S.E.M., n=10-32 cells per data point.

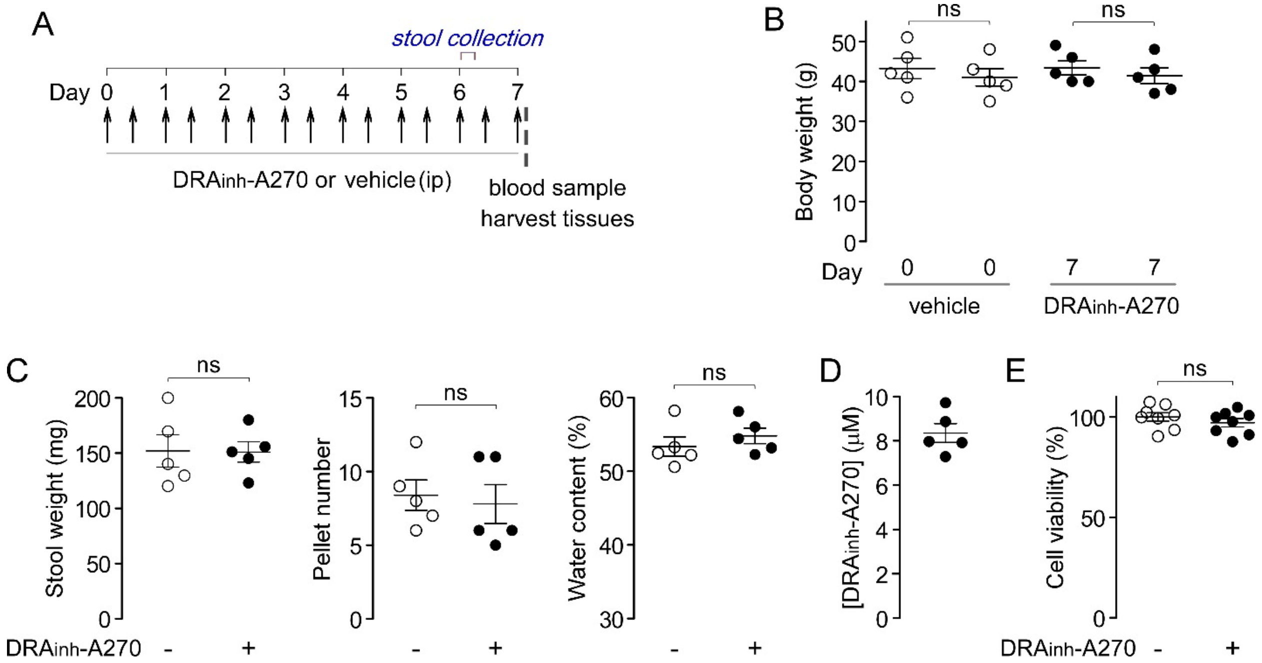


**Supplementary Figure 6. Toxicity and off-target studies in mice. A.** Experimental protocol. **B.** Effect of 7-day DRA$_{inh}$-A270 (10 mg/kg, ip, twice daily) or vehicle treatment on body weight, n=5 mice per group. **C.** 3-hour stool weight, pellet number and water content at Day 6 in mice treated with DRA$_{inh}$-A270 or vehicle per panel A, n=5 mice per group. **D.** DRA$_{inh}$-A270 concentration in plasma at 3 h after the final DRA$_{inh}$-A270 dose in mice treated for 1 week as in A, n=5 mice. **E.** In vitro cytotoxicity of DRA$_{inh}$-A270 (10 μM with 24 h incubation) measured by Alamar Blue assay in FRT cells, 0.1% DMSO was used as vehicle (negative) control. N=8 wells per group. As a positive control 20% DMSO reduced cell viability to 16±0.6 % (not shown). Mean ± S.E.M., unpaired t-test, ns: not significant.

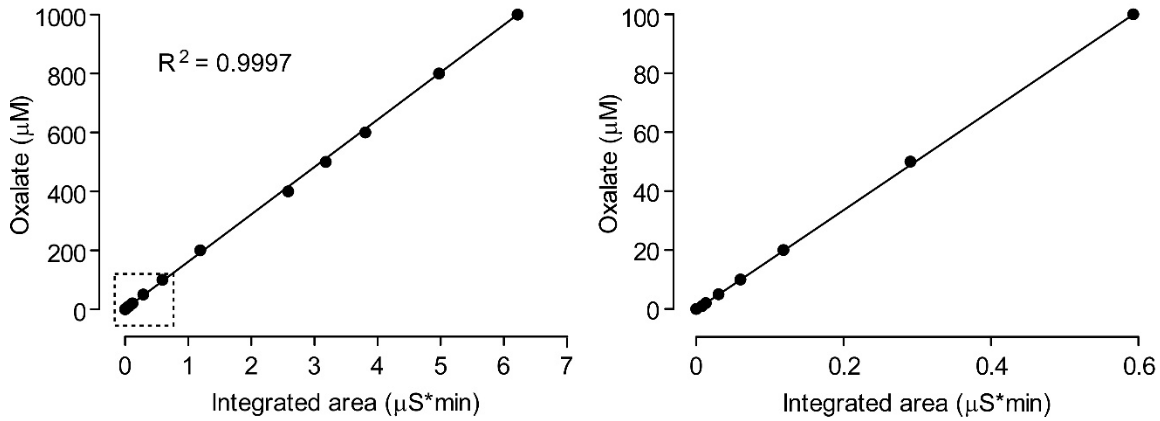


**Supplementary Figure 7. Oxalate standard curve in aqueous solution.** Integrated area of oxalate peak (μS*min) for aqueous solutions (phosphate-buffered saline) containing specified concentrations of sodium oxalate. The graph on the right is an expansion of the dashed rectangular area on the left.

**Supplementary Table 1.** Eurofins SafetyScreen44 panel results for $DRA_{inh}$-A270. Assays were done in duplicate with 10 μM $DRA_{inh}$-A270, mean % inhibition is shown.

| Assay name | Inhibition (%) |
|---|---|
| 5-HT1A Human Serotonin GPCR Binding (Agonist Radioligand) Assay | 0 |
| 5-HT1B Human Serotonin GPCR Binding (Antagonist Radioligand) Assay | -1 |
| 5-HT2A Human Serotonin GPCR Binding (Antagonist Radioligand) Assay | 17 |
| 5-HT2B Human Serotonin GPCR [$^3$H]LSD Binding (Agonist Radioligand) Assay | 64 |
| 5-HT3 Human Serotonin Ion Channel Binding (Antagonist Radioligand) Assay | -6 |
| A2A Human Adenosine GPCR Binding (Agonist Radioligand) Assay | 46 |
| Acetylcholinesterase Human Enzymatic Assay | 10 |
| alpha1A Human Adrenoceptor GPCR Binding (Antagonist Radioligand) Assay | 5 |
| alpha2A Human Adrenoceptor GPCR Binding (Antagonist Radioligand) Assay | 47 |
| AR Human Androgen NHR Binding (Agonist Radioligand) Assay | 13 |
| beta1 Human Adrenoceptor GPCR Binding (Antagonist Radioligand) Assay | 14 |
| beta2 Human Adrenoceptor GPCR Binding (Antagonist Radioligand) Assay | 3 |
| Cav1.2 (L-type) Rat Calcium Ion Channel Binding (Dihydropyridine Site) Assay | -6 |
| CB1 Human Cannabinoid GPCR Binding (Antagonist Radioligand) Assay | -3 |
| CB2 Human Cannabinoid GPCR Binding (Agonist Radioligand) Assay | -1 |
| CCK1 (CCKA) Human Cholecystokinin GPCR Binding (Agonist Radioligand) Assay | -5 |
| COX1 Human Cyclooxygenase Enzymatic Assay | -1 |
| COX2 Human Cyclooxygenase Enzymatic Assay | 15 |
| D1 Human Dopamine GPCR Binding (Antagonist Radioligand) Assay | 13 |
| D2S Human Dopamine GPCR Binding (Antagonist Radioligand) Assay | 2 |
| DAT Human Dopamine Transporter Binding (Antagonist Radioligand) Assay | -2 |
| delta (DOP) Human Opioid GPCR Binding (Antagonist Radioligand) Assay | -3 |
| ETA Human Endothelin GPCR Binding (Agonist Radioligand) Assay | 13 |
| Glutamate (NMDA, Non-Selective) Rat Ion Channel [$^3$H] CGP-39653 Binding Assay | 16 |
| GR Human Glucocorticoid NHR Binding (Agonist Radioligand) Assay | -7 |
| H1 Human Histamine GPCR Binding (Antagonist Radioligand) Assay | 27 |
| H2 Human Histamine GPCR Binding (Antagonist Radioligand) Assay | -2 |
| hERG Human Potassium Ion Channel [$^3$H] Dofetilide Binding (Antagonist Radioligand) Assay | 19 |
| kappa (KOP) Human Opioid GPCR Binding (Antagonist Radioligand) Assay | 7 |
| KV (Non-Selective) Rat Potassium Ion Channel [$^{125}$I] alpha-Dendrotoxin Binding Assay | -1 |
| M1 Human Acetylcholine (Muscarinic) GPCR Binding (Antagonist Radioligand) Assay | 9 |
| M2 Human Acetylcholine (Muscarinic) GPCR Binding (Antagonist Radioligand) Assay | -3 |
| M3 Human Acetylcholine (Muscarinic) GPCR Binding (Antagonist Radioligand) Assay | 8 |
| Monoamine Oxidase A (MAO-A) Human Enzymatic Assay | 73 |
| mu (MOP) Human Opioid GPCR Binding (Antagonist Radioligand) Assay | -5 |
| nAChR (alpha4/beta2) Human Ion Channel Binding (Agonist Radioligand) Assay | -3 |
| NET Human Norepinephrine Transporter Binding (Antagonist Radioligand) Assay | 29 |
| Non-Selective Rat GABAA Ion Channel [$^3$H] Flunitrazepam Binding (Agonist Radioligand) Assay | -8 |
| Non-Selective Rat Sodium Ion Channel [$^3$H] Batrachotoxinin Binding (Site 2) Assay | 22 |
| PDE3A Human Phosphodiesterase Enzymatic Assay | 4 |
| PDE4D2 Human Phosphodiesterase Enzymatic Assay | -2 |
| SET Human Serotonin Transporter Binding (Antagonist Radioligand) Assay | -7 |
| V1A Human Vasopressin / Oxytocin GPCR Binding (Antagonist Radioligand) Assay | -1 |

**Supplementary Table 2.** CYP450 inhibition by $DRA_{inh}$-A270 showing mean inhibition for each isoform. CYP450 activity was measured in human liver microsomes in the presence and absence of 10 μM $DRA_{inh}$-A270 (n=2 per condition). Substrates for each isoform: CYP1A2: phenacetin (30 μM); CYP2C9: diclofenac (10 μM); CYP2C19: S-mephenytoin (35 μM); CYP3A4: midazolam (5 μM) and testosterone (80 μM); CYP2D6: bufuralol (10 μM); CYP2C8: paclitaxel (10 μM); CYP2B6: bupropion (70 μM). Positive controls (reference) were: CYP1A2: α-maphthoflavone; CYP2C9: sulfaphenazole; CYP2C19: omeprazole; CYP3A4: ketoconazole; CYP2D6: quinidine; CYP2C8: nicardipine; CYP2B6: clopidogrel.

| Compound | % of CYP inhibition (mean) | | | | | | | |
|---|---|---|---|---|---|---|---|---|
| | 1A2 | 2B6 | 2C8 | 2C9 | 2C19 | 2D6 | 3A4 (midazolam) | 3A4 (testosterone) |
| Reference | 100 | 95.6 | 97.1 | 92.8 | 96 | 83.5 | 98.4 | 95.5 |
| $DRA_{inh}$-A270 | 11.4 | 55.6 | 49.9 | 18.2 | 5.3 | 24.3 | -20.5 | -11.9 |

**Supplementary Table 3.** hERG activity. hERG activity (n≥2 per condition) was measured by manual patch-clamp in Chinese Hamster Ovary cells stably transfected with hERG. Cisapride (0.1 μM) was used as positive control Mean ± S.D., n=2-4 experiments per group.

| **Compound** | **% hERG inhibition** |
|---|---|
| $DRA_{inh}$-A270 | 1.9 ± 4.6 |
| cisapride | 93.9 ± 0.4 |

**Supplementary Table 4.** Microsomal stability of $DRA_{inh}$-A270 in mouse, rat and human liver microsomes. $DRA_{inh}$-A270 was incubated in the reaction mixture with NADPH for 0, 5, 15, 30 and 45 min at 37°C in duplicate with final liver microsomal protein concentration of 0.5 mg/mL. $DRA_{inh}$-A270 concentration was measured at each time point by LC-MS/MS and in vitro elimination half-life was calculated. Ketanserin was used as the reference compound with rapid metabolism.

| Test compound | Species | Percent remaining (%) | | | | | $T_{1/2}$ (minutes) |
|---|---|---|---|---|---|---|---|
| | | 0 min | 5 min | 15 min | 30 min | 45 min | |
| ketanserin | human | 100 | 88.4 | 69.1 | 49.4 | 39.9 | 33.40 |
| | rat | 100 | 68.9 | 36.5 | 16.1 | 6.9 | 11.86 |
| | mouse | 100 | 82.1 | 54.4 | 30.8 | 19.5 | 18.94 |
| $DRA_{inh}$-A270 | human | 100 | 94.9 | 102.5 | 95.7 | 102.5 | ∞ |
| | rat | 100 | 97.9 | 91.8 | 96.3 | 95.1 | 876.17 |
| | mouse | 100 | 99.1 | 96.5 | 84.1 | 88.8 | 201.23 |

**Supplementary Table 5.** CBC, serum electrolytes, anion gap and chemistries in mice (on regular diet) treated with $DRA_{inh}$-A270 (10 mg/kg) or vehicle twice daily for 7 days. Mean ± S.E.M., unpaired t-test, n=5 mice per group.

| | **vehicle** | **$DRA_{inh}$-A270** | **P value** |
|---|---|---|---|
| Hemoglobin (g/dL) | 14.6±0.1 | 14.8±0.2 | >0.05 |
| Hematocrit (%) | 55.7±0.4 | 56.8±0.8 | >0.05 |
| WBC ($10^3$/µL) | 7.4±1.2 | 6.6±1.7 | >0.05 |
| Platelets ($10^3$/µL) | 739±21 | 812±36 | >0.05 |
| Sodium (mmol/L) | 156.0±0.5 | 154.6±0.6 | >0.05 |
| Potassium (mmol/L) | 5.1±0.4 | 4.8±0.1 | >0.05 |
| Chloride (mmol/L) | 118±0.3 | 116±0.7 | >0.05 |
| Bicarbonate $TCO_2$ (mmol/L) | 15±1.1 | 16±1.0 | >0.05 |
| Anion gap (mmol/L) | 22±1.9 | 23±1.6 | >0.05 |
| Calcium (mg/dL) | 8.3±0.1 | 8.4±0.1 | >0.05 |
| Phosphorus (mg/dL) | 7.2±0.4 | 6.9±0.4 | >0.05 |
| Creatinine (mg/dL) | 0.04±0.02 | 0.06±0.02 | >0.05 |
| BUN (mg/dL) | 15±0.9 | 13±1.3 | >0.05 |
| Total protein (g/dL) | 4.5±0.02 | 4.4±0.1 | >0.05 |
| Albumin (g/dL) | 2.5±0.04 | 2.4±0.05 | >0.05 |
| Globulin (g/dL) | 2.0±0.02 | 2.0±0.04 | >0.05 |
| Total bilirubin (mg/dL) | 0.3±0.03 | 0.3±0.03 | >0.05 |
| Bilirubin - unconjugated (mg/dL) | 0.1±0 | 0.08±0.2 | >0.05 |
| Bilirubin - conjugated (mg/dL) | 0.20±0.03 | 0.22±0.02 | >0.05 |
| Glucose (mg/dL) | 175±7.8 | 156±9.7 | >0.05 |
| ALT (U/L) | 56±14 | 40±7 | >0.05 |
| AST (U/L) | 83±4 | 76±11 | >0.05 |
| ALP (U/L) | 65±5 | 70±4 | >0.05 |
| Creatine kinase (U/L) | 603±72 | 431±96 | >0.05 |
| Cholesterol (mg/dL) | 112±5 | 98±10 | >0.05 |
